# Qualitative evaluation of antimicrobial use with gyssens method in the internal medicine wards of Adam Malik General Hospital Medan

**DOI:** 10.1017/ash.2025.163

**Published:** 2025-09-03

**Authors:** Restuti Hidayani Saragih, Lenni Evalena Sihotang, Fandy Ong Jaya, Terang Meliala, Otneil Karnianta Keliat, Lily Sherly Hasugian, Mario Eka Paskah Sinullinga, Maulinda Putri, Junita Tarigan, Franciscus Ginting

**Affiliations:** 1 Division of Tropical Medicine and Infectious Diseases, Department of Internal Medicine, Faculty of MedicineUniversitas Sumatera Utara, Indonesia; 2 Antimicrobial Stewardship Team, Adam Malik Hospital, Medan, Indonesia; 3 Faculty of MedicineUniversitas Sumatera Utara, Indonesia; 4 Faculty of MedicineUniversitas HKBP Nomensen, Indonesia; 5 Mitra Sejati Hospital, Medan, Indonesia; 6 Sidikalang General Hospital, Dairi, Indonesia; 7 Adam Malik Hospital, Medan, Indonesia; 8 Infection Prevention and Control Committee, Adam Malik Hospital, Medan, Indonesia

**Keywords:** antimicrobial resistance, gyssens method, qualitative evaluation

## Abstract

**Objective:** Antimicrobial resistance will become one of the most lethal conditions, which will raise burdens in many sectors across the One Health spectrum. The irrational use of antibiotics without proper monitoring is one of the causes of antimicrobial resistance. The quality of antimicrobial usage could be evaluated quantitatively using the Defined Daily Dose (DDD) method and qualitatively using the Gyssens method. This study aims to evaluate the antimicrobial usage qualitatively. **Method:** This is a retrospective study using data from the electronic medical record of Adam Malik Hospital within the period of July 2022–December 2023. All adult patients (>18 years old) in the internal medicine ward with a history of antimicrobial prescriptions were included in this study. The quality evaluation was carried out by the researchers, of whom three of them are members of the hospital’s antimicrobial stewardship program (PPRA) team. **Result:** There are 293 cases of antimicrobial use included in this study. Most of the population in this study was female (51.9%), in the 18–59 year group (74.1%), and respiratory tract infection was mostly diagnosed in this study (31.2%). Based on the Gyssens analysis, only 33% of cases of antimicrobial usage were appropriate (category 0). It means more than half of the antimicrobial use in internal medicine wards were irrational. **Conclusion:** The rationality of antimicrobial use is one of the most important ways to reduce the rate of antimicrobial resistance. This study shows only 33% of cases of appropriate antimicrobial usage, which is relatively low. Because irrational antimicrobial use can lead to antimicrobial resistance, prolonged length of stay and mortality, efforts need to be taken to improve the quality of antimicrobial use.

